# Promoted Relationship of Cardiovascular Morbidity with Air Pollutants in a Typical Chinese Urban Area

**DOI:** 10.1371/journal.pone.0108076

**Published:** 2014-09-23

**Authors:** Ling Tong, Kai Li, Qixing Zhou

**Affiliations:** 1 School of Environmental Science and Engineering, Tianjin University, Tianjin, China; 2 Department of Industrial Engineering, Nankai University, Tianjin, China; 3 Key Laboratory of Pollution Process and Environmental Criteria (Ministry of Education), College of Environmental Science and Engineering, Nankai University, Tianjin, China; The Ohio State University, United States of America

## Abstract

**Background:**

A large number of studies about effects of air pollutants on cardiovascular mortality have been conducted; however, those investigating association between air pollutants and cardiovascular morbidity are limited, especially in developing countries.

**Methods:**

A time-series analysis on the short-term association between outdoor air pollutants including particulate matter (PM) with diameters of 10 µm or less (PM_10_), sulfur dioxide (SO_2_) and nitrogen dioxide (NO_2_) and cardiovascular morbidity was conducted in Tianjin, China based on 4 years of daily data (2008–2011). The morbidity data were stratified by sex and age. The effects of air pollutants during the warm season and the cool season were also analyzed separately.

**Results:**

Each increase in PM_10_, SO_2_, and NO_2_ by increments of 10 µg/m^3^ in a 2-day average concentration was associated with increases in the cardiovascular morbidity of 0.19% with 95 percent confidence interval (95% CI) of 0.08–0.31, 0.43% with 95% CI of 0.03–0.84, and 0.52% with 95% CI of −0.09–1.13, respectively. The effects of air pollutants were more evident in the cool season than those in the warm season, females and the elderly were more vulnerable to outdoor air pollution.

**Conclusions:**

All estimated coefficients of PM_10_, SO_2_ and NO_2_ are positive but only the effect of SO_2_ implied statistical significance at the 5% level. Moreover, season, sex and age might modify health effects of outdoor air pollutants. This work may bring inspirations for formulating local air pollutant standards and social policy regarding cardiovascular health of residents.

## Introduction

As the largest harbor in northern China, Tianjin is a fast-growing and economically developed city. It has an area of approximately 11,919 km^2^ and a population of 10 million. A continental monsoonal climate featuring hot and humid summer, and dry and cold winter is typical here. The mean annual temperature and precipitation is 13.1°C and 389.4 mm, respectively. It is wetter in summer than in winter. The domestic heating season is generally between November and March. The average wind speeds range from 16 to 24 km/h. A comprehensive industrial system including integrated machinery, electronics, petroleum and chemicals, metallurgy, textiles and vehicles has been built in this city. The industrial prosperity brings about severe air pollution problems. From 2008 to 2011, both daily concentrations of PM_10_ and SO_2_ (80.2 µg/m^3^ and 148.1 µg/m^3^, respectively) exceeded the Class I levels of the Chinese national standards (daily average: 50 µg/m^3^ for both PM_10_ and SO_2_) [1] and the standards of the World Health Organization (WHO) (daily average: 50 µg/m^3^ and 24 µg/m^3^ for PM_10_ and SO_2_, respectively) [2]. Air pollution exerts tremendous burdens to public health, ranking as the 13th leading cause of mortality [3]–[5].

There are numerous studies of similar questions that have been conducted in North America and Western Europe. Several years ago, the Health Effects Institute sponsored some studies under the acronym PAPA (http://pubs.healtheffects.org/types.php?type=1 for published reports), including "Public Health and Air Pollution in Asia (PAPA): Coordinated Studies of Short-Term Exposure to Air Pollution and Daily Mortality in Two Indian Cities" and "Public Health and Air Pollution in Asia (PAPA): Coordinated Studies of Short-Term Exposure to Air Pollution and Daily Mortality in Four Cities". Pathophysiologic mechanisms of air pollutant-induced cardiovascular morbidity and mortality are also being studied widely [6]–[8], besides, some studies on the associations between air pollutants and cardiovascular diseases have been conducted in terms of epidemiology [9]–[13]. However, investigations on the relationship between air pollutants and cardiovascular morbidity are scarce at present [14], especially in Asian countries where, arguably, both living conditions and health indicators may be quite different.

This study presents an investigation of this issue in a typical Chinese city to increase our understanding of cardiovascular health risks associated with polluted air and provide some scientific basis for establishment of public health and environmental protection policies [15].

## Materials and Methods

### 2.1. Subject Data

Daily air pollution data on PM_10_, SO_2_ and NO_2_ were obtained from the website of the Tianjin Environmental Monitoring Centre. Daily mean temperature and relative humidity were obtained from the China Meteorological Data Sharing Service System (Data S1).

Daily cardiovascular morbidity data from 1 January 2008 to 31 November 2011 was collected from the Centers for Disease Control and Prevention of Urban Districts in Tianjin, China (Nankai, Heping, Hexi, Hedong and Hongqiao districts), covering around 77000 local residents (SI). Information of patients including those under the age of 18 was anonymized and were validated each year by China CDC. They were coded according to the ICD-10 (the 10th revision of International Classification of Diseases) and classified into cardiovascular causes including cerebral infarction, primary diagnosed hypertension, cerebral hemorrhage, acute myocardial infarction and subarachnoid hemorrhage [16]. They were also stratified by sex and age (0–18, 18–44, 45–64, and ≥65 years) [17]. The ethical committee of the coordinating center of five urban CDCs in Tianjin approved the study (Full name is "CDC biomedical ethics council").

Air pollutant concentrations and meteorological measures including temperature and humidity are shown in Table 1 and 2.

**Table 1:** Description of air pollutants and meteorological parameters during 2008–2011 in Tianjin.

Season	PM_10_	SO_2_	NO_2_	Temperature (°C)	Humidity (%)
Warm season (n = 732)	79.1±1.3	11.8±1.3	6.2±0.4	22.5±0.2	61.2±0.6
Cool season (n = 698)	78.7±1.6	142.5±4.0	21.2±0.8	3.5±0.3	52.0±0.7
Entire period (n = 1430)	78.9±1.1	75.6±2.9	13.5±0.5	13.2±0.3	56.7±0.5

**Table 2:** Quartile values of three pollutants.

Quartile	PM_10_ (µg/m^3^)	SO_2_ (µg/m^3^)	NO_2_ (µg/m^3^)
5th	29.4	11.3	0.1
25th	58.0	62.4	5.9
50th	73.6	119.1	13.7
75th	97.0	215.5	25.3
95th	149.8	368.5	50.5

### 2.2. Statistical methods

All analyses were conducted with statistical software package SAS version 9.1. Time-series analysis was utilized to explore modification effects of season, age and sex on the association between air pollutants and morbidity in Tianjin from 2008 to 2011. In detail, the generalized linear model with natural splines (ns) functions of time, weather conditions accommodate nonlinear and non-monotonic relationships of morbidity with time, temperature and relative humidity were utilized for the analysis [17]. In the basic model, morbidity outcomes were included without air pollution variables. Residuals of the basic models were examined to determine whether there were discernible patterns and autocorrelation by means of residual plots and partial autocorrelation function (PACF) plots. Day of the week (DOW) was considered as a dummy variable in the basic modes. After the establishment of the basic model, PM_10_ and covariates (temperature, humidity, and SO_2_ and NO_2_ concentrations) were introduced into it and their effects were analyzed. The selection of df (degrees of freedom) for time trends was done with the PACF [17], [18]. 4 df per year for time trends were used in our basic models for cardiovascular morbidity. In addition, we used 3 df (during whole period of study) for temperature and humidity. This modeling procedure was carried out for each series studied and the core models were assessed with plots of model residuals and fitted values as well as plots of the estimated partial autocorrelation functions.

The estimated pollution log-relative rate β is obtained through fitting of the following log-linear generalized linear model:

logE(Yt)  =  βZt + DOW + ns(time, df) + ns(temperature/humidity, 3) + intercept (1)

Where E(Yt) and β represent the expected morbidity numbered at day t and the log-relative rate of morbidity corresponding to a unit increase of air pollutants, respectively; Zt indicates pollutant concentrations at day t; DOW is dummy variable for day of the week; ns (time, df) is the ns function of calendar time with 4 df to adjust for seasonality and other time-varying influences on admissions (e.g. influenza epidemics and longer-term trends); and ns (temperature/humidity, 3) is the ns function for temperature and humidity with 3 df.

The data were stratified by sex and age. We analyzed the associations for the warm season (April-September), the cool season (October-March) and the entire year, respectively (Kan et al., 2008b). The basic models of seasonal analyses were different from those of the whole-period in terms of df for time trends. The effects were quantified on the basis of the percentage change in risk per 10 mg/m^3^ increase in the concentration of each pollutant. The statistical significance was defined as *p*<0.05.

## Results

### 3.1. Time series plot of the morbidity, exposure-response relationships, ACF and PACF of morbidity

**Figure 1:**
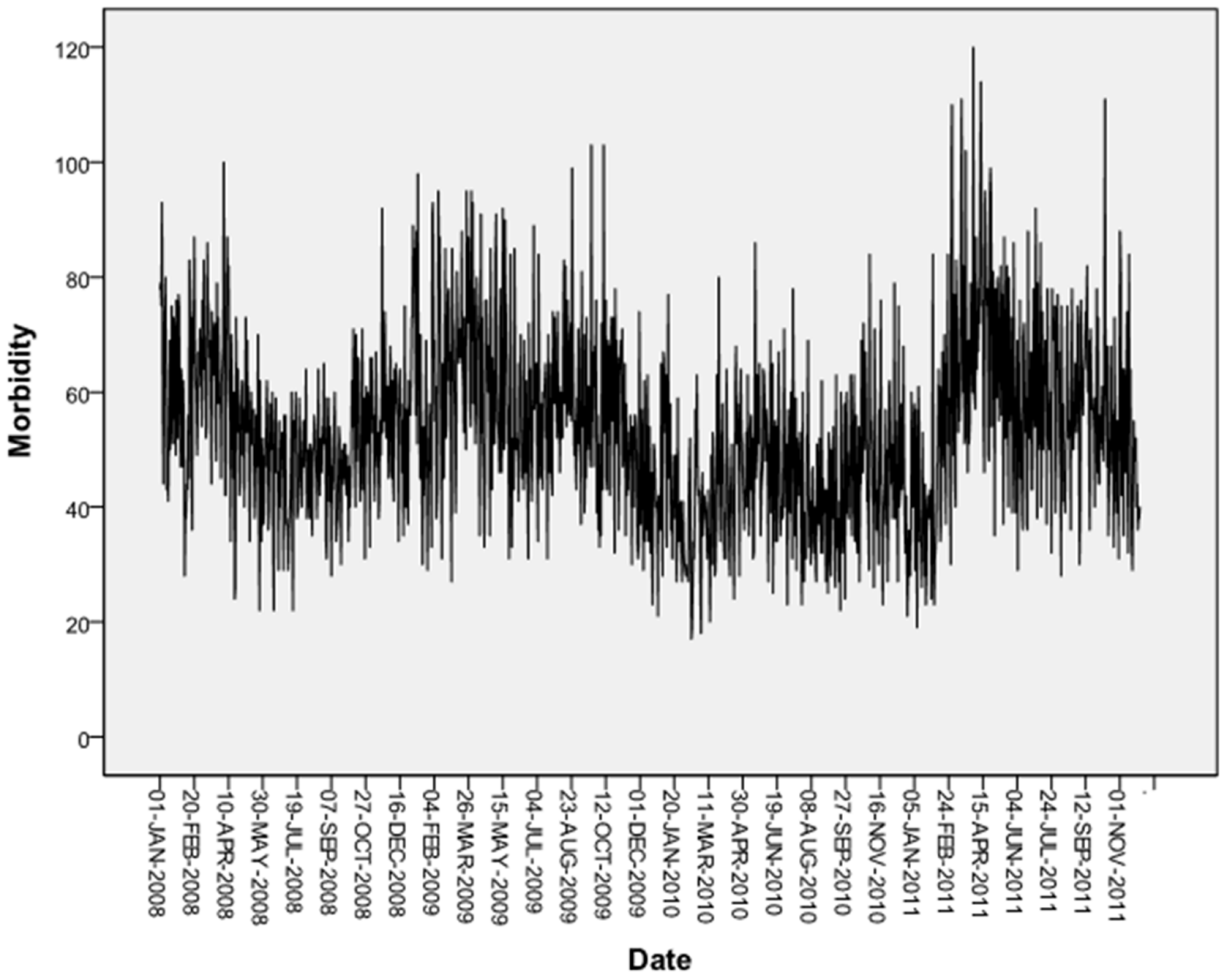
Time series of cardiovascular morbidity from 2008 to 2011 in Tianjin.

**Figure 2:**
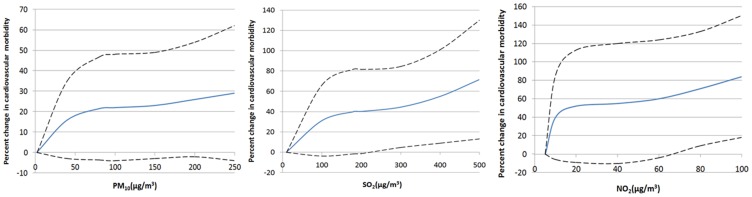
Exposure-response relationships (smoothing plots) of air pollutants against cardiovascular morbidity. The x-axis is the pollutant concentrations; the y-axis is the estimated percent change in cardiovascular mortality; the solid blue lines indicate the estimated mean percent change in daily mortality outcomes with the dashed lines representing the 95% CI.

**Figure 3:**
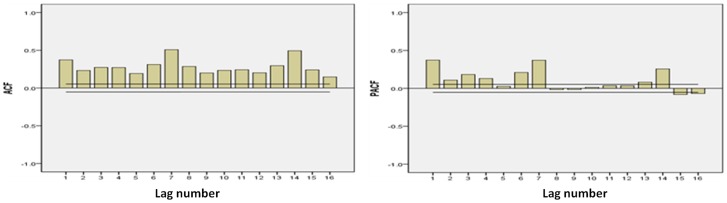
ACF and PACF of cardiovascular morbidity (log) and (log+difference). The x-axis is the lag number.

**Figure 4:**
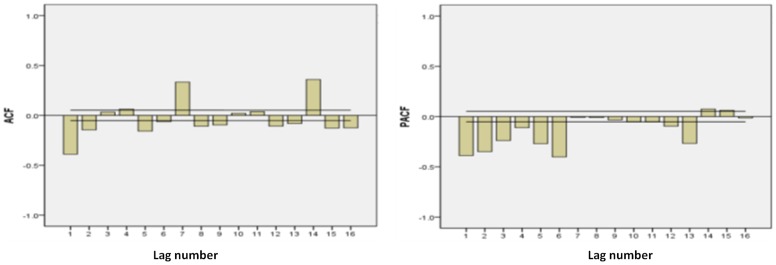
ACF and PACF of residual after modeling (basic model). The x-axis is the lag number.

**Figure 5:**
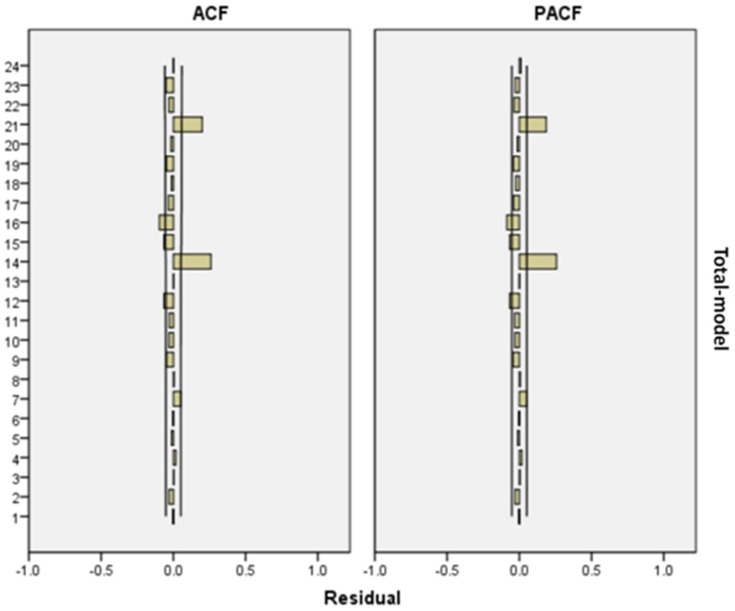
ACF and PACF of residual after modeling (basic model).

The time series plot of cardiovascular morbidity from 2008 to 2011 in Tianjin is shown in Fig. 1. Fig. 2 demonstrates the exposure–response relationships for PM_10_, SO_2_ and NO_2_ (2-day moving average of air pollutant concentration (lag 01)) with cardiovascular mortality during 2008–2011 in Tianjin. In Fig. 3, ACF and PACF of cardiovascular morbidity are depicted as original data (log). ACF and PACF of cardiovascular morbidity as original data (log+Difference) are shown in Fig. 4. Fig. 5 gives ACF and PACF of residual after modeling (basic model).

### 3.2. Effects by sexes and ages

As shown in Table 3, the percent increases associated with air pollutant concentrations varied by sex or age groups. The effect estimates of pollutants among females were greater than those among males, especially the estimate for NO_2_ was thrice as much as that among males, although their between-sex differences were insignificant statistically. The effects among those ≥65 were greater than those in the other two groups.

**Table 3:** Mean daily morbidity and percent increase (95% CI) in cardiovascular morbidity of Tianjin residents associated with a 10-µg/m^3^ increase in air pollutant concentrations by sex and age^a^.

	Mean daily morbidity	Pollutant		
		PM_10_	SO_2_	NO_2_
Sex				
Female	21.86±0.23	0.25 (0.10, 0.39) <0.001	0.47 (0.10, 0.85) 0.019	0.57 (0.07, 1.06) 0.022
Male	31.52±0.29	0.13 (0.01, 0.25) 0.042	0.39 (0.21, 0.58) <0.001	0.17 (−0.16, 0.50) 0.892
Age				
5–44	1.94±0.04	0.07 (−0.05, 0.21) 0.687	0.11 (−0.02, 0.24) 0.566	0.42 (−0.19, 1.03) 0.601
45–64	20.27±0.19	0.12 (−0.16, 0.39) 0.844	0.21 (−0.10, 0.53) 0.947	0.26 (0.02, 0.51) 0.009
≥65	31.17±0.27	0.20 (0.09, 0.30) <0.001	0.47 (0.09, 0.85) 0.001	0.53 (0.31, 0.76) <0.001

a Current day temperature and humidity (lag 0) and 2-day moving average of air pollutant concentration (lag 01) with 3df of temperature and humidity were used.

### 3.3. Effects by seasons

The daily average morbidity in the warm and cool season was 51.8±0.5 and 50.9±0.6, respectively. For the entire period, the average value was 51.4±0.4 and an increase of 10 µg/m^3^ of 2-day average concentration of PM_10_, SO_2_ and NO_2_ correspond to 0.19% (95% CI, 0.08–0.31), 0.43% (95% CI, 0.03–0.84) and 0.52% (95% CI, −0.09–1.13) increases, respectively (Table 4).

**Table 4:** Mean percent increase (95% CI) of cardiovascular morbidity outcomes associated with 10-µg/m^3^ increase in air pollutant concentrations by season, 2008–2011^a^.

Pollutant	Warm season	Cool season	Entire period
PM_10_	0.15 (−0.19, 0.50) *p* = 0.869	0.24 (0.20, 0.28)* *p*<0.001	0.19 (0.08, 0.31) *p* = 0.016
SO_2_	0.24 (0.01, 0.47) *p* = 0.046	0.66 (0.35, 0.97)* *p* = 0.001	0.43 (0.03, 0.84) *p* = 0.037
NO_2_	0.21 (−0.20, 0.63) *p* = 0.667	0.71 (0.15, 1.27) *p* = 0.033	0.52 (−0.09, 1.13) *p* = 0.424

a We used current day temperature and humidity (lag 0) and 2-day moving average of air pollutant concentration (lag 01), and applied 3df to temperature and humidity.

*Significantly different from the warm season (*p*<0.05).

Sensitivity analyses results for PM_10_ as shown in Table 5 and Table 6. They showed that the results were fairly robust for various concentrations, specifications for temperature, methods of aggregating daily data, df used in the smoothers, and alternative spline models.

**Table 5:** Sensitivity analyses for varying degrees of freedom for time trend and weather conditions: df = 4–7 per year for time trend (A), df = 3–6 for current day temperature/relative humidity (B)^a^.

	A			
	4	5	6	7
Warm season	0.153 (−0.191, 0.498)	0.154 (−0.190, 0.499)	0.156 (−0.189, 0.502)	0.158 (−0.187, 0.504)
Cool season	0.242 (0.203, 0.280)	0.242 (0.203, 0.281)	0.244 (0.204, 0.283)	0.245 (0.205, 0.285)
Entire period	0.194 (0.076, 0.311)	0.195 (0.078, 0.313)	0.197 (0.078, 0.315)	0.199 (0.080, 0.318)

	B			
	3	4	5	6
Warm season	0.153 (−0.191, 0.498)	0.153 (−0.191, 0.498)	0.153 (−0.192, 0.498)	0.152 (−0.192, 0.497)
Cool season	0.242 (0.203, 0.281)	0.242 (0.203, 0.281)	0.242 (0.203, 0.281)	0.242 (0.202, 0.281)
Entire period	0.194 (0.076, 0.311)	0.194 (0.076, 0.311)	0.194 (0.076, 0.311)	0.193 (0.076, 0.310)

a We used 2-day moving average (lag 01) of PM_10_ concentration, and current day temperature and humidity (lag0).

**Table 6:** Sensitivity analyses for two different spline models^a^.

	Quadratic spline	Cubic nature spline
Warm season	0.151 (−0.194, 0.495)	0.153 (−0.191, 0.498)
Cool season	0.251 (0.190, 0.311)	0.242 (0.203, 0.281)
Entire period	0.196 (0.078, 0.315)	0.194 (0.076, 0.311)

a Current day temperature and humidity (lag 0) and 2-day moving average of PM_10_ (lag 1) are used, 3 df for temperature and humidity is applied.

## Discussion

PM_10_ in developed countries are mainly discharged from various automobiles including large amount of secondary organic aerosols. In Tianjin, ground dust, vehicle, cement dust and incineration are the primary PM_10_ sources [19]. Coal and crude oil are responsible for 66% and 30% of energy supply, respectively [20]. Crude oil dominated by PM with fine fractions carries more toxic substance and is easier to penetrate into the circulation system of humans than coal. The main components of other PM sources such as raise dust and sand dust are less toxic inorganic minerals.

Generally speaking, the smoking habit exerted much more oxidative and inflammatory influences on males than air pollution [21]. In China, smoking is much more prevalent among men than women [22], this may have affected male health to a greater degree than environmental factors, therefore we suspect females were more sensitive to smoking than males. As for age, older people ≥65 were more vulnerable to air pollution (especially SO_2_) than people in the other two age groups. In terms of their occupation, percent increase of myocardial infarction exposed to welding and soldering fumes in the Copenhagen male study population was 1.1 (95%CI, 0.6–2.2) [23], much higher than the effect estimates on males in this study (Table 3). This is mainly attributed to the higher concentration PM exposure with more toxicity in the workplace than public environment.

Though not measured in this study, other factors such as underlying diet could also have affected our observed results. Because nutrients with natural chelating properties, including antioxidants, herbs, minerals, essential amino acids and fiber, can detoxify human bodies, they can protect humans from oxidative stress of free radicals derived from air pollutants to some extent. Vitamins C, E and A in majority of plant and fish foods favored by residents in Tianjin as an important coastal city can interfere with or scavenge reactive oxygen species within cells [24]–[26]. To better understand the modification effects of living habits, socioeconomic and demographic factors on the associations, more individual features (smoking and eating habit, occupation, education attainment levels, physical activity, socioeconomic status, etc.) should be taken into consideration in the future.

As for seasonal effects, some studies indicated that high temperatures in the warm season and low temperatures in the cold season are associated with increased cardiovascular mortality [17], [27]–[29]. It is likely that they can also affect cardiovascular morbidity. Therefore, morbidity data were stratified by the warm and cool season defined as April-September and the cool season defined as October-March, respectively. Therefore, this research also demonstrated that the effect estimates of air pollutants in Tianjin might be modified by seasons. Exposure patterns may contribute to this season-specific observation. Personal exposure was reduced by decreased time spent outdoors caused by heavy rain. In contrast, people are more likely to go outdoors and open windows in the cool season with the drier and less variable in Tianjin. The effect estimates of SO_2_ and NO_2_ in the cool season were much higher than those in the warm season. Significant associations were observed for PM_10_ and SO_2_ in the cool season (0.24 (0.20, 0.28) and 0.66 (0.35, 0.97), respectively), while the effect of SO_2_ in the warm season was insignificant. It may be caused by coal combustion accounting for the largest proportion of heat supply in the cool season. Besides, the significant Pearson correlation coefficient between PM_10_ and SO_2_ (0.197, *p*<0.01) might partially explain this phenomenon. NO_2_ was mainly released from vehicle emission and partially from coal combustion, which may account for the insignificant relationship between NO_2_ and morbidity. The constituents of the complex mix of PM_10_ may vary by seasons, different from the gaseous pollutants (SO_2_ and NO_2_).

There are some limitations in our analyses. Ozone was not included in our studies due to a lack of monitoring. Our ability to separate the independent effect of each pollutant was limited by high correlations between PM and gaseous pollutants including SO_2_ and NO_2_ in Tianjin. Moreover, the role of each specific component of air pollution should be examined to determine the combination of particles responsible for the increases in environment-induced health concerns. These investigations are paramount for policy makers to carry out improved interventions that will impact health hazards related with air pollutants, especially on the increased risks of cardiovascular morbidity.

## Conclusions

In summary, we found that PM_10_ and SO_2_ were significantly associated with cardiovascular morbidity in the cool season in Tianjin in this time-series analysis. The mean percent increases (95% CI) of cardiovascular morbidity outcomes per 10 µg/m^3^ increase in PM_10_ and SO_2_ concentrations by season were 0.24 (0.20, 0.28) and 0.66 (0.35, 0.97), respectively. Besides modification effect of a season, individual features (sexes, ages) might also interfere with the effect estimates of air pollutants. Females were more sensitive than males to air pollutants. The effects of air pollutants on elder people (≥65) were greater than those <65. These data can enable policy makers to enforce or improve existing legislation that control air pollution after weighing the disadvantages of potentially slowing rapid economic development.

## Supporting Information

Data S1 **Cardiovascular, air pollutants and meteorological measurements data.**
(XLS)
