# Correction: Promoted Relationship of Cardiovascular Morbidity with Air Pollutants in a Typical Chinese Urban Area

**DOI:** 10.1371/journal.pone.0118427

**Published:** 2015-02-17

**Authors:** The PLOS ONE Staff

In the Funding section, the grant number for the Ministry of Education, People’s Republic of China grant is incorrect. Please refer to the correct Funding section here.

This research was financially supported by The National Natural Science Foundation as a key project (grant No. 21037002), and by the Ministry of Education, People’s Republic of China as an innovative research team project (grant No. IRT13024). The funders had no role in study design, data collection and analysis, decision to publish, or preparation of the manuscript.
